# Cultural sexual selection in monogamous human populations

**DOI:** 10.1098/rsos.160946

**Published:** 2017-06-21

**Authors:** Wataru Nakahashi

**Affiliations:** School of Advanced Sciences, SOKENDAI (The Graduate University for Advanced Studies), Shonan Village, Hayama, Kanagawa 240-0193, Japan

**Keywords:** cultural evolution, runaway process, success bias, conformist bias, modern behaviour, fashion cycle

## Abstract

In humans, both sexes sometimes show peculiar mating preferences that do not appear to increase their fitness either directly or indirectly. As humans may transmit their preferences and target culturally, and these may be artificially modifiable, I develop theoretical models where a preference and/or a trait are culturally transmitted with a restriction of the trait modification. I assume a monogamous population where some individuals fail to find a mate, and this affects the preference and the trait in the next time step. I show that a strong aversion to, or high tolerance of, failed individuals are necessary for the evolution of irrational preferences that neither seek good genes nor any direct benefit. This evolution is more likely to occur when the preference and/or the trait are cultural rather than genetic. These results may partly explain why humans sometimes show mating preferences for exaggerated physical and cultural traits.

## 1. Introduction

Many mating behaviours and the associated physical traits are considered to have evolved through sexual selection. The most studied are the exaggerated male traits, such as the plumage of the peacock, as well as the female mating preferences for such traits. The theory of sexual selection was first proposed by Darwin [1], who argued that exaggerated male traits could evolve if female mating preferences led to more *charming* males, with exaggerated traits having more mates and more offspring, but he failed to explain why such preferences were adaptive. Fisher [2] suggested that a joint evolution of female mating preference and male trait would occur because of a genetic correlation of the two; this is known as the ‘runaway process’ or the ‘sexy son hypothesis’ [3], where females who prefer attractive males may produce attractive sons with a higher fitness than non-attractive males and, thus, the female preference for the attractive male trait increases in frequency. Zahavi [4] considered that males take the risk of displaying exaggerated traits to advertise their quality and females prefer more exaggerated males, who will produce higher quality offspring; this is the ‘handicap hypothesis’. Empirical studies and theoretical models have tested these hypotheses and confirmed that sexual selection may have played an important role in the evolution of many animal traits [5–7].

However, theoretical models of sexual selection for non-human animals do not apply to humans without modification because of the unique characteristics of human mating behaviour. Although many human behaviours are unique, this study focuses on only three characteristics: monogamy, male mating preference and cultural transmission. As some animals also display these characteristics, how each one affects the sexual selection process has partly been studied, in theory, but there are no models that simultaneously assume these three characteristics.

Although polygynous marriage is not totally prohibited in many (traditional) human societies, there are limited numbers of males with more than one wife, and the number of wives is often limited [8]. Strong monogamous marriage norms have spread across Westernized societies, probably because cultural evolution has favoured normative monogamy [9]. Humans have smaller testes than chimpanzees, which form promiscuous multi-male–multi-female groups [10], implying that human (hominid) societies have long been semi-monogamous [11,12]. In monogamous species, attractive (popular) males have little reproductive advantage because they mate with, at most, one female, thus limiting the number of offspring, and choosy females are maladaptive because they have to compete intensely with other females for attractive males who may have many candidates as a mate, suggesting that exaggerated male traits hardly evolve through sexual selection compared with polygynous species. In fact, theoretical models have shown that exaggerated male traits may evolve only when more attractive males can mate earlier, when there is a shortage of available female partners, or when variance in female quality is large, i.e. only when attractive males have a sufficiently large advantage [13,14]. In short, previous theoretical models have suggested that sexual selection by female choice has not strongly influenced the evolution of human-male traits compared with polygynous species.

As the number of offspring is limited in (mammalian) females (the ‘Bateman's principle’ [15]), attractive (popular) females have little reproductive advantage, and choosy males are maladaptive because of intense intrasexual competition for attractive females, resulting in exaggerated female traits hardly evolving through sexual selection compared with exaggerated male traits. In fact, males are generally more decorative than females in many polygynous species, although there is increasing evidence of male mate choice for female traits [16–18]. As the situation is similar to monogamous male traits, theoretical models have shown that exaggerated female traits may evolve (i.e. a runaway process may occur) when there is a shortage of available male partners or when the variance in male quality is large [14,19]. Moreover, there must be a disagreement between the fertility optimum and the viability optimum of the female trait for male choosiness to evolve, and then males tend to prefer more fertile females who have more *feminine* traits (less resemblance to males), because choosy males can obtain a direct benefit through mating with more fertile females [19]. These theoretical results may explain many human-male mating preferences for more feminine female traits such as larger breasts [20], a lower waist-to-hip ratio [21], a more feminized face [22] and lighter-than-average skin colour [23], although whether these preferences are adaptive is controversial [24]. However, although most male mating preferences may be associated with female fertility, males sometimes show seemingly irrational preferences, which is difficult to explain based on previous theoretical models.

Human behaviour is often influenced by culture, which is one of our most unique characteristics and forms the basis of our great prosperity. It may be difficult to identify a human complex behaviour that is completely independent of culture or social learning. Human mating behaviour is no exception; e.g. we dress in fashionable clothes to attract the opposite sex. Foot binding in ancient China and neck rings in the Kayan are extreme examples of this. Since so-called modern behaviour is often characterized by artistic ornaments, such as shell beads and the use of ochre [25–27], mating behaviours of early modern humans and possibly Neanderthals may have been influenced by cultural transmission, although the evolutionary cause and actual uses of these cultural traits remain controversial [28–34]. As cultural preferences are affected by our social learning tendencies and cultural traits are artificially modifiable, their coevolutionary processes should differ greatly from the genetic processes. Although some theoretical models have assumed cultural preferences [35,36] and traits [37–39], they have considered the coevolution of female preference and male trait in polygynous species. Therefore, these models do not explain why male preferences for exaggerated female traits sometimes evolve in monogamous human populations.

Here, I formulate and analyse models of sexual selection in monogamous species where a trait and a preference are continuously distributed and culturally transmitted. I assume that some individuals do not have a mate during each breeding season because of a shortage of available mating partners, and this affects the expression of trait and preference in the next time step (generation). I also consider the case when a trait or a preference is genetically transmitted. I assume that the mating system of the population is completely monogamous to ensure that the model applies to both female preferences for male traits and male preferences for female traits (but the model may apply to polygynous mating systems with minor modifications; see Discussion). Although a monogamous human population is implicitly assumed, the model may also apply to other animals, such as monogamous birds with culturally transmitted songs that attract the opposite sex. The model may also apply to human fashion cycles.

## 2. Model

### 2.1. Cultural trait and cultural preference

In the first model, we assume that both a mating preference and a target trait are culturally transmitted. The preference, *y*, is expressed in only a ‘choosy sex’ and the trait, *z*, is present in only a ‘decorative sex.’ Here, we do not specify which sex (male or female) is choosy or decorative because completely monogamous species are considered. The assumption of the mating system is explained later.

On an appropriate scale of measurement, the preference and the (matured) trait are assumed to have normal distributions, *p*(*y*) and *t*(*z*), with the means $\bar{y}$ and $\bar{z}$, and variances *τ*^2^ and *σ*^2^, respectively. Formally, the distribution of the preference is

2.1 $$p(y)=\frac{1}{\sqrt{2\pi }\tau }\;\exp\;\left[-{\frac{\left(y-\bar{y}\right)}{2\tau^{2}}}^{2}\right]$$

and that of the trait is

2.2 $$t(z)=\frac{1}{\sqrt{2\pi }\sigma }\;\exp\;\left[-{\frac{\left(z-\bar{z}\right)}{2\sigma^{2}}}^{2}\right].$$

The mating preference of a choosy individual with preference *y* for a decorative individual with trait *z* is denoted as ψ(z|y). In this model, we assume the absolute preference [40],

2.3 $$\psi (z|y)\propto \exp\;\left[-\frac{{\left(z-y\right)}^{2}}{2\nu^{2}}\right].$$

This implies that a *y* choosy individual prefers a decorative individual with trait value of *y*, and the preference decreases as the deviation from *y* increases. The choosiness is milder when *ν*^2^ is larger.

The following mating system is assumed. In each breeding season, individuals of the decorative sex display their traits to attract individuals of the choosy sex. Each choosy individual observes the traits of decorative individuals and courts one individual depending on his/her mating preference. Each decorative individual randomly selects one mating partner from the choosy individuals who court him/her, but if no individuals court him/her, he/she has no mates during that breeding season. Changes seen in the results when some individuals can have multiple mates will be discussed later.

Then the probability that a choosy individual with preference *y* courts a decorative individual with trait *z* relative to all the decorative individuals is

2.4 $$\psi^{*}(z|y)=\frac{\psi (z|y)}{\int t(z)\psi (z|y)\text{d}z}.$$

So, the relative number of choosy individuals courting a decorative individual with trait *z* is

2.5 $$U(z)=\int p(y)\psi^{*}(z|y)\text{d}y.$$

In other words, *U*(*z*) is the ‘popularity’ of a *z* individual. Denoting the number of individuals of the choosy sex relative to that of the decorative sex in the breeding season as *r* (>0), the expected number of choosy individuals courting a *z* individual is *rU*(*z*). Provided each choosy individual decides on his/her courting target independently of other choosy individuals, the probability that a *z* individual is approached by 0, 1, 2, 3 … individuals follows a Poisson distribution with a mean of *rU*(*z*). Therefore, the probability that a *z* individual has a mate is

2.6 π(z)=1−exp [−rU(z)].

Assuming that decorative individuals differ little in their popularity, i.e. U(z)≈1, we can approximate

2.7 $$\pi (z)\approx 1-(1+r){\text{e}}^{-r}+r{\text{e}}^{-r}U(z).$$

This approximation is justified in appendix A.

Individuals of the decorative sex can be classified into two groups, those with and those without a mate in the breeding season. These are the ‘popular’ and ‘unpopular’ individuals, respectively. The average trait of popular individuals (popular trait) is

2.8 $${\bar{z}}_{M}=\frac{\int z\pi (z)t(z)\text{d}z}{\int \pi (z)t(z)\text{d}z}\approx (1-sR)\bar{z}+sR\bar{y}$$

and that of unpopular individuals (unpopular trait) is

2.9 $${\bar{z}}_{L}=\frac{\int z[1-\pi (z)]t(z)\text{d}z}{\int [1-\pi (z)]t(z)\text{d}z}\approx (1+sr)\bar{z}-sr\bar{y},$$

where

2.10 $$s=\frac{\sigma^{2}}{\nu^{2}+\sigma^{2}}$$

and

2.11 $$R=\frac{r{\text{e}}^{-r}}{1-{\text{e}}^{-r}}.$$

The proportion of popular individuals is

2.12 $$P_{M}=\int \pi (z)t(z)\text{d}z\approx 1-{\text{e}}^{-r}$$

and that of unpopular individuals is

2.13 $$P_{L}=\int [1-\pi (z)]t(z)\text{d}z\approx {\text{e}}^{-r}.$$

Individuals of the decorative sex in the next generation (or the next breeding season) observe the traits of popular and unpopular individuals and gain their ‘ideal’ traits. Assuming that the ideal trait of each individual is the weighted mean of the observed popular and unpopular traits, the ideal trait of individual *i* is

2.14 $$z_{I}^{i}=a{\bar{z}}_{M}^{i}+(1-a){\bar{z}}_{L}^{i},$$

where ${\bar{z}}_{M}^{i}$ and ${\bar{z}}_{L}^{i}$ are the average popular and unpopular traits observed by individual *i*, respectively. Parameter *a* describes the effect of each average trait (*a* > 0). When 0 < *a* ≤ 1, as *a* increases, the ideal trait approaches the average popular trait from the average unpopular trait. When *a* > 1, as *a* increases, the ideal trait deviates from the average popular trait to the opposite side of the average unpopular trait. In other words, parameter *a* describes an aversion to unpopular individuals.

Although all individuals observe the traits in the same population, they may sample different individuals and incur an observation error. Therefore, the ideal traits for decorative individuals may distribute normally as

2.15 $$\varphi (z_{I})=\frac{1}{\sqrt{2\pi }\kappa }\;\exp\;\left[-{\frac{\left(z_{I}-{\bar{z}}_{I}\right)}{2\kappa^{2}}}^{2}\right],$$

where *κ* reflects the sampling and observation errors. A small *κ* also implies a strong conformist bias in social learning, because conformity should decrease the variance.

If there is a restriction to decorate (modify) the trait, the difficulty increases as the deviation from the plain trait *θ* (the easiest trait to express) increases. Formally, the ease with which to express *z* is

2.16 $$w(z)\propto \exp\;\left[-{\frac{\left(z-\theta \right)}{2\omega^{2}}}^{2}\right].$$

The restriction is weaker when *ω*^2^ is larger. In other words, each individual originally (genetically) expresses trait *θ* and must make an effort to attain the ideal trait (in §2.3, we consider a model in which the genetic trait also coevolves). This restriction is similar to viability selection in genetic sexual selection models. As shown in appendix B, assuming $\kappa^{2}\omega^{2}/(\kappa^{2}+\omega^{2})=\sigma^{2}$, the average trait in the next generation (breeding season) is

2.17 $${\bar{z}}^{\prime }=\theta +q\{1-s[aR-(1-a)r]\}(\bar{z}-\theta )+qs[aR-(1-a)r](\bar{y}-\theta ),$$

where

2.18 $$q=\frac{\omega^{2}}{\kappa^{2}+\omega^{2}}.$$

Note that the average ideal trait is the average trait of decorative individuals when $a=P_{M}=r/(r+R)$.

Similarly, individuals of the choosy sex in the next generation (breeding season) observe the traits of popular and unpopular individuals and gain the ideal traits for courting. Note that the preferences of choosy individuals are invisible, so only the traits of the decorative sex affect the preferences in the next generation. As humans sometimes show so-called mate choice copying [41], and a popular mate is sometimes regarded as a status symbol, choosy individuals may prefer popular traits rather than unpopular ones. Here, we assume that choosy individual *i* has the ideal trait to court as

2.19 $$y_{I}^{i}=b{\bar{z}}_{M}^{i}+(1-b){\bar{z}}_{L}^{i},$$

where *b* describes his/her aversion to unpopular individuals (*b* > 0). Even if a choosy individual prefers unpopular traits to avoid competition with other choosy individuals, this equation is applicable if we consider a small value for *b*.

In contrast with the trait expression, there is no restriction in preference, e.g. choosy individuals can prefer ‘Prince Charming’. Therefore, as shown in appendix B, assuming that sampling and observation errors entail variance *τ*^2^, the average preference in the next generation (breeding season) is

2.20 $${\bar{y}}^{\prime }=\theta +\{1-s[bR-(1-b)r]\}(\bar{z}-\theta )+s[bR-(1-b)r](\bar{y}-\theta ).$$

Note that the average trait of decorative individuals is ideal for choosy individuals when b=r/(r+R).

From (2.17) and (2.20), a recursive matrix can be drawn

2.21 $$\left(\begin{array}{c} {\bar{z}}^{\prime }-\theta \\ {\bar{y}}^{\prime }-\theta \end{array}\right)=\left(\begin{array}{cc} q\{1-s[aR-(1-a)r]\} & qs[aR-(1-a)r]\\ 1-s[bR-(1-b)r] & s[bR-(1-b)r] \end{array}\right)\left(\begin{array}{c} \bar{z}-\theta \\ \bar{y}-\theta \end{array}\right).$$

The equilibrium is $\left(\bar{z},\bar{y})=(\theta ,\theta \right)$, i.e. average decorative individuals show the easiest trait to express, and this trait is the most preferred by average choosy individuals. The equilibrium is stable when and only when

2.22*a* $$\begin{array}{rl} b & <\frac{1+sr}{s(R+r)}, \end{array}$$

2.22*b* $$\begin{array}{rl} b & <a+\frac{1}{qs(R+r)} \end{array}$$

**Figure 1.**
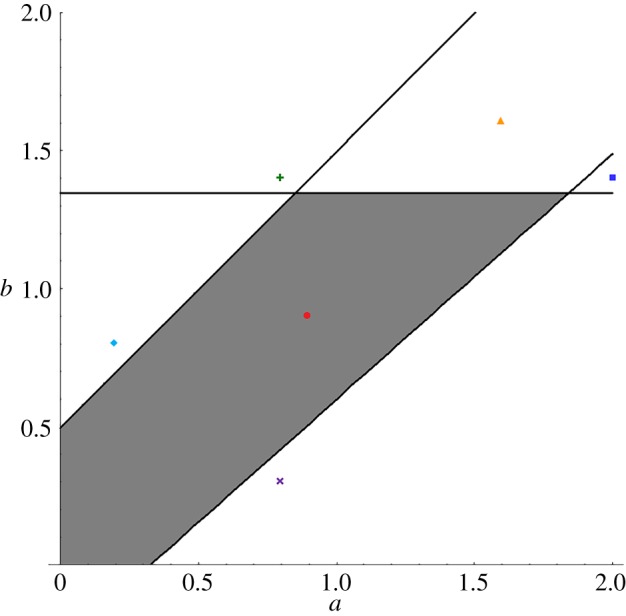
The stability condition of the equilibrium is shown in the (*a*,*b*)-parameter space. The equilibrium is stable in the area covered by the grey pentagon. Each symbol indicates the parameter condition of (*a*,*b*) in figure 2. Parameters are *r* = 3.0, *s* = 0.8 and *q* = 0.8.

**Figure 2.**
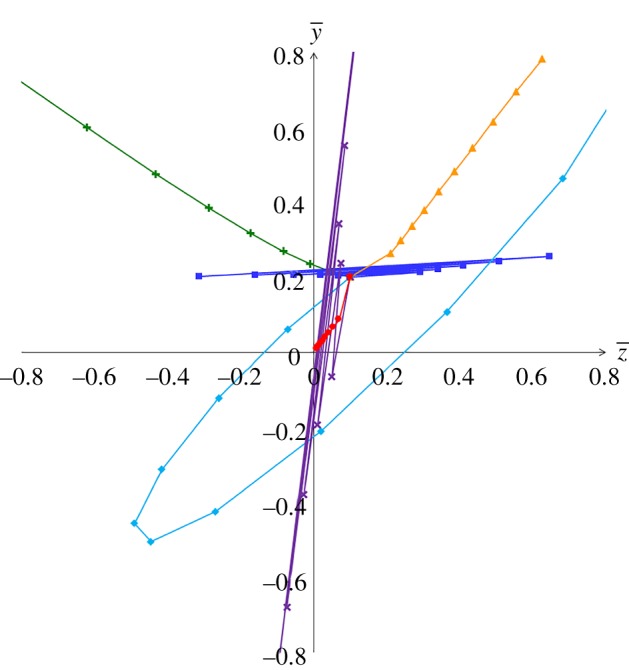
The evolutionary trajectories (generation 0–10) from the initial state $\left(\bar{z},\bar{y})=(0.1,0.2\right)$ under several (*a*,*b*)-parameter conditions are shown in the $\left(\bar{z},\bar{y}\right)$-parameter space. The parameter conditions of each trajectory are (*a*, *b*) = (0.9, 0.9), (0.8, 1.4), (2.0, 1.4), (0.2, 0.8), (0.8, 0.3) and (1.6, 1.6), respectively, which are shown in figure 1. Parameters are *r* = 3.0, *s* = 0.8, *q* = 0.8 and *θ* = 0.0.

2.22*c* $$\begin{array}{rl} \;\mathrm{and}\;b & >\frac{2q}{1+q}a-\frac{1+q-sr(1-q)}{s(1+q)(R+r)} \end{array}$$

are satisfied. Figure 1 shows the area where the equilibrium is stable in the (*a*,*b*)-parameter space, and figure 2 shows the evolutionary trajectories under several conditions in the $\left(\bar{z},\bar{y}\right)$-parameter space. When choosy individuals show a sufficiently strong aversion to unpopular individuals (large *b*), the equilibrium is unstable regardless of the properties of decorative individuals, and exaggerated traits and preferences evolve eventually. In this case, the equilibrium is more likely to be unstable as *r* increases when *s* ≤ 1/2. Even when 0 ≤ *a* ≤ 1 and 0 ≤ *b* ≤ 1, i.e. when both choosy and decorative individuals have the ideal trait that lies between the average popular and unpopular traits, the equilibrium can be unstable when

2.23*a* qs(R+r)>1

or

2.23*b* $$s\left(r+\frac{2q}{1+q}R\right)>1,$$

i.e. when the restriction of trait modification is weak (large *q*), the choosiness is strong (large *s*) and the relative number of choosy individuals is large (large *r*). However, as shown in appendix C, the equilibrium is stable provided both choosy and decorative individuals have the ideal trait that lies between the average trait and the average popular trait, i.e. $\bar{z}\leq {\bar{z}}_{I}\leq {\bar{z}}_{M}$ and $\bar{z}\leq {\bar{y}}^{\prime }\leq {\bar{z}}_{M}$ if $\bar{z}\leq {\bar{z}}_{M}$, or ${\bar{z}}_{M}\leq {\bar{z}}_{I}\leq \bar{z}$ and ${\bar{z}}_{M}\leq {\bar{y}}^{\prime }\leq \bar{z}$ if ${\bar{z}}_{M}<\bar{z}$.

### 2.2. Genetic trait and cultural preference

Next, we consider a cultural mating preference for a genetic trait. The fitness (relative number of offspring) of a decorative individual without a mate in the breeding season (initially) is 1 – *c* (0 < *c* ≤ 1), i.e. even if he/she has a mate later on, the number of offspring is smaller than an individual with a mate acquired earlier, because of the late mating. Then, as shown in appendix D, the average trait of decorative individuals in the next generation before viability selection is

2.24 $${\bar{z}}_{I}=\bar{z}+\frac{c{\text{e}}^{-r}(1-{\text{e}}^{-r})}{1-c{\text{e}}^{-r}}({\bar{z}}_{M}-{\bar{z}}_{L})\frac{h_{t}^{2}}{2},$$

where $h_{t}^{2}$ is the (narrow-sense) heritability of the trait ($0<h_{t}^{2}\leq 1$). When the viability of a *z* individual is

2.25 $$w(z)\propto \exp\;\left[-{\frac{\left(z-\theta \right)}{2\omega^{2}}}^{2}\right],$$

the model is equivalent to the previous model if we denote

2.26 $$a=(1-{\text{e}}^{-r})\left(1+\frac{c{\text{e}}^{-r}}{1-c{\text{e}}^{-r}}\cdot \frac{h_{t}^{2}}{2}\right).$$

Then, as $1-{\text{e}}^{-r}<a<1$, i.e. $\bar{z}<{\bar{z}}_{I}<{\bar{z}}_{M}$ or ${\bar{z}}_{M}<{\bar{z}}_{I}<\bar{z}$, the equilibrium $\left(\bar{z},\bar{y})=(\theta ,\theta \right)$ is stable, provided choosy individuals have the ideal trait that lies between the average trait and the average popular trait, i.e. $\bar{z}\leq {\bar{y}}^{\prime }\leq {\bar{z}}_{M}$ or ${\bar{z}}_{M}\leq {\bar{y}}^{\prime }\leq \bar{z}$.

### 2.3. Genetic–cultural trait and cultural preference

Third, we consider a cultural mating preference for a trait that is affected by both genes and culture. For example, skin colour is genetically transmitted, but it can be (slightly) modified by avoiding sunlight or by applying powder/cream. In this model, we assume the order of life-history events such as (i) birth, (ii) genetic trait expression, (iii) viability selection, (iv) trait modification, (v) mating and (vi) reproduction. The distribution of genetic trait (after viability selection) is

2.27 $$g(x)=\frac{1}{\sqrt{2\pi }\rho }\;\exp\;\left[-{\frac{\left(x-\bar{x}\right)}{2\rho^{2}}}^{2}\right]$$

and the viability of an *x* individual is

2.28 $$v(x)\propto \exp\;\left[-{\frac{\left(x-\theta \right)}{2\xi^{2}}}^{2}\right].$$

Assuming a restriction to modify the trait, the ease of expression of trait *z* from trait *x* is

2.29 $$w(z)\propto \exp\;\left[-{\frac{\left(z-x\right)}{2\omega^{2}}}^{2}\right].$$

Other assumptions remain the same as in the first and second models. Then, as shown in appendix E, the equilibrium $\left(\bar{z},\bar{y},\bar{x})=(\theta ,\theta ,\theta \right)$ is stable, provided both choosy and decorative individuals have the ideal trait that lies between the average trait and the average popular trait, i.e. $\bar{z}\leq {\bar{z}}_{I}\leq {\bar{z}}_{M}$ and $\bar{z}\leq {\bar{y}}^{\prime }\leq {\bar{z}}_{M}$ if $\bar{z}\leq {\bar{z}}_{M}$, or ${\bar{z}}_{M}\leq {\bar{z}}_{I}\leq \bar{z}$ and ${\bar{z}}_{M}\leq {\bar{y}}^{\prime }\leq \bar{z}$ if ${\bar{z}}_{M}<\bar{z}$.

### 2.4. Cultural trait and genetic preference

Fourth, we consider a genetic mating preference for a cultural trait with assumptions as in the second model. As shown in appendix F, the average preference of individuals in the next generation is

2.30 $${\bar{y}}^{\prime }=\bar{y}+\frac{\left(1-{\text{e}}^{-r})(1-c{\text{e}}^{-r}-cr{\text{e}}^{-r}\right)}{r(1-c{\text{e}}^{-r})}({\bar{z}}_{M}-{\bar{z}}_{L})\frac{h_{p}^{2}}{2},$$

where $h_{p}^{2}$ is the (narrow-sense) heritability of the preference ($0<h_{p}^{2}\leq 1$). The model is equivalent to the first model if we denote

2.31 $$b=(1-{\text{e}}^{-r})\left(1+\frac{1}{sr}-\frac{1-c{\text{e}}^{-r}-cr{\text{e}}^{-r}}{r(1-c{\text{e}}^{-r})}\frac{h_{p}^{2}}{2}\right).$$

Then, as $1-{\text{e}}^{-r}<b<(1+sr)\text{/}s(R+r),$ the equilibrium $\left(\bar{z},\bar{y})=(\theta ,\theta \right)$ is stable, provided decorative individuals possess the ideal trait lying between the average trait and the average popular trait, i.e. $\bar{z}\leq {\bar{z}}_{I}\leq {\bar{z}}_{M}$ or ${\bar{z}}_{M}\leq {\bar{z}}_{I}\leq \bar{z}$ (see appendix F).

### 2.5. Genetic trait and genetic preference

Fifth, we consider a genetic mating preference for a genetic trait, which is equivalent to [19]. In this case, although both $1-{\text{e}}^{-r}<a<1$ and $1-{\text{e}}^{-r}<b<(1+sr)\text{/}s(R+r)$ are always satisfied, the equilibrium $\left(\bar{z},\bar{y})=(\theta ,\theta \right)$ can be unstable because of the genetic covariance between the preference and the trait, which can be regarded as a runaway process. As shown in [19], a runaway process is more likely to occur when *ω*^2^ is large, *ν*^2^ is small, $h_{t}^{2}$ and $h_{p}^{2}$ are large and *r* is small, i.e. when viability selection is weak, choosiness is strong, heritability of the trait and the preference are large, and the relative number of choosy individuals is small. Interestingly, the effect of the relative number of choosy individuals on the stability of the equilibrium is opposite to that in the cultural model.

## 3. Discussion

Ever since the time of Darwin [1], human sexual selection has been hotly debated but, unfortunately, many arguments are theoretically invalid and some are simply wrong. For example, although sexual selection should increase the sexual dimorphism of the target trait [19], sexual selection theory has been applied to many sexually monomorphic human traits (abilities), such as bipedalism, intelligence, creativity, language, art and music [42–44]. Many arguments have also implicitly assumed ‘everlasting’ mating preferences for target traits and have not explained the evolution of these preferences, i.e. the same mistake made by Darwin [1] initially. As mate choice cannot evolve if it is costly without compensating benefits [45], and both male and female choosiness should be costly in semi-monogamous human populations because of mate competition, a direct or indirect benefit of choosiness that counterbalances cost is necessary for the evolution of preferences. Although choosy individuals may obtain a benefit if the trait honestly signals the bearers' quality, costly preferences never evolve unless there is a mechanism that maintains the honesty of signals, such as resistance to parasites [46], mutation bias [45] and a disagreement between fertility and viability optimums of the trait [19]. Therefore, even if a human-specific trait seems to contribute to mate acquisition, it cannot be concluded that the trait evolved through sexual selection. Nevertheless, we cannot simply consider that sexual selection has had little effect on human evolution because human behaviour is often culturally transmitted, which may change the results of general sexual selection theories that assume genetic traits and preferences.

In this study, I have theoretically analysed sexual selection models where a trait and/or a preference are culturally transmitted. Although humans adopt various social learning strategies [47], the most often observed tendencies may be success bias (copying successful individuals) and conformist bias (copying the majority), which may have evolved as an adaptation to temporally and spatially varying environments [48,49]. As these biases are also observed in human mating preferences [41,50], it is assumed that each individual of the choosy and decorative sexes uses the information about who succeeded or failed to find a mate during the breeding season, and adopts the weighted average trait as the ideal trait. Success bias is strong when an aversion to unsuccessful individuals is strong (large *a* and *b*) and conformist bias is strong when the trait and the preference distribute narrowly (small *σ*^2^ and *τ*^2^). It has also been assumed that there is a restriction on modifying the plain (genetic) trait (i.e. the easiest trait to express). Note that when a trait is culturally transmitted, reproductive success of individuals does not directly affect the generational change of the trait, but we can regard the process as sexual selection because mating success of individuals has the main influence on the trait evolution.

The presence of a unique equilibrium has been demonstrated where both the average trait of the decorative sex and the most preferable trait for the choosy sex are plain. Therefore, when this equilibrium is stable, the trait never evolves to be exaggerated. In other words, exaggerated traits and peculiar preferences can be observed only when sexual selection destabilizes the equilibrium favoured by natural (non-sexual) selection. As expected, when the restriction of trait modification is weak (*ω*^2^ is large) and choosiness is strong (*ν*^2^ is small), the equilibrium is more likely to be unstable. However, the equilibrium is always stable, provided that both the choosy and decorative sexes have the ideal trait that lies between the average trait and the average popular trait (average trait of individuals with a mate). In other words, a strong aversion to, or high tolerance of, unsuccessful individuals is necessary for exaggerated traits and peculiar preferences to evolve through cultural sexual selection. This result is unchanged even if we assume that either the trait or the preference is genetically transmitted.

The most important difference between genetic and cultural transmission is the restriction on the range of trait and preference values in the next generation (time step). When the trait or the preference is genetically transmitted, it should lie within a certain range in the next generation, while there is no restriction in cultural transmission. Therefore, we can suppose that genetic traits and preferences are less likely to be exaggerated (peculiar) compared with cultural traits. In humans, peculiar mating preferences, such as those for small feet (foot binding) or long necks (neck rings), are sometimes observed, and these may be transmitted culturally. On the other hand, many globally observed mating preferences, such as male preferences for female traits indicating reproductive capacity, are probably genetic (instinctive) and can be regarded as the equilibrium in genetic models [19]. In short, irrational preferences may seldom evolve genetically but sometimes arise culturally and, therefore, it is important to dismiss the preconception that every human mating preference aims at gaining good genes or a direct benefit.

Humans often give greater weight to negative entities than positive ones, known as ‘negativity bias’ [51]. This bias may cause the coevolution of exaggerated traits and peculiar preferences because a strong aversion to unsuccessful individuals destabilizes the equilibrium. In other words, once a trait accidentally becomes unpopular, it would be more unpopular in the next time step because individuals dislike unpopular traits, which favours exaggerated traits that are dissimilar to the unpopular trait. It should be noted that this acceleration occurs regardless of any learning bias of the decorative sex if the choosy sex has a sufficiently strong aversion to unpopular traits.

When individuals of the choosy sex are *realists* and prefer the unpopular traits to avoid mate competition with other individuals (*b* is small), the equilibrium can also be unstable. In this case, the trait and the preference oscillate to become exaggerated (figure 2). A similar destabilization occurs when the decorative sex has *sympathy* for unsuccessful individuals and thus expresses similar traits to them (*a* is small). In these cases, the destabilization is more likely to occur when the opposite sex prefers popular traits, i.e. disagreement between male and female ideal traits is important for destabilization. Interestingly, the destabilization can occur even when both choosy and decorative sexes have the ideal trait that lies between the average popular and unpopular traits (i.e. 0 ≤ *a* ≤ 1 and 0 ≤ *b* ≤ 1). In other words, even when male and female ideal traits are not extreme, the joint acceleration of trait and preference can occur if there is a large sexual difference in ideal traits. In humans, male preferences are often incomprehensible to females and vice versa [52,53], which may lead to the emergence of exaggerated traits and peculiar preferences. This situation may also apply to human fashion cycles, where fluctuations in popularity of cultural traits are often observed and may be caused by positive and negative preferences for major cultural traits [54].

Although the model assumes a completely monogamous population, this assumption can easily be relaxed to apply the model to a polygynous mating system. When females are choosy, and assuming that each male accepts every female who courts him, the mating system becomes one of polygyny. Then, if males with at least one mate and males without a mate are regarded as popular and unpopular, respectively, the model does not essentially change, provided that both the trait and the preference are culturally transmitted. Even if the trait or the preference is genetically transmitted, the model is unchanged if males with multiple mates have a smaller number of offspring by one mate than males with only one mate (this affects only parameter *c*). When males are choosy, and assuming that each male courts multiple (a fixed number of) females and each female randomly selects a mate from the males who court her, this is a polygynous mating system, but the model is essentially unchanged, provided that every female with a mate has the same number of offspring (even this provision is unnecessary when both the trait and the preference are culturally transmitted). In this case, we may redefine parameter *r* as the product of the adult sex ratio (male/female) and the number of females each male courts. It should be noted that the number of females without a mate may be smaller than that of males without a mate in polygynous populations, provided that the adult sex ratio is almost one, i.e. *r* is larger when males are choosy than when females are choosy. Therefore, we may suppose that exaggerated female cultural traits are more likely to evolve through sexual selection than those of the male because the equilibrium is more likely to be unstable when *r* increases. This may explain cultural evolution of female costly body modifications such as foot binding and neck rings. In short, although the model assumes monogamy, it is applicable to polygynous populations if minor modifications are made.

In the model, exaggerated traits are more likely to evolve when success bias is strong (*a* and *b* are large) and conformist bias is weak (*σ*^2^ is large), which is similar to cultural evolution models [30,32,55,56] where strong success bias and weak conformist bias accelerate cultural evolution. This implies that our social learning strategy has strongly influenced cultural evolution regardless of the role of cultural traits. Therefore, if we assume that the emergence of modern behaviour was caused by a change in hominid social learning strategies (abilities), we may explain why functional tools, such as microliths, and artistic ornaments, such as shell beads, were associated with modern behaviour. The present model also shows that a strong aversion to unsuccessful individuals leads to the emergence of exaggerated traits, which is also similar to previous cultural evolution models [30,32] that suggested that a strong aversion to Neanderthal culture may have caused the so-called ‘artistic explosion’ in European Upper Palaeolithic modern humans, i.e. certain artistic behaviours of modern humans emerged first in Europe rather than Africa [57]. In short, although the model considers cultural sexual selection, the results may apply to various cultural traits that are not associated with mating behaviours.

In conclusion, when the trait and the preference are culturally transmitted, they are more likely to be exaggerated (peculiar) compared with when they are genetically transmitted. However, when there is a strong restriction of trait modification, decorative individuals express the plain (genetic) trait, which is the most preferred by choosy individuals. A strong aversion to, or high tolerance of, individuals without a mate is necessary for exaggerated traits and peculiar preferences to coevolve. Although the model assumes cultural transmission of trait and preference in monogamous populations, it is applicable to genetic transmission and polygynous populations following the implementation of some minor modifications.

## Data accessibility

This study does not use any data.

## Appendix A

From (2.1) to (2.5), we have

A 1 $$U(z)=\frac{\sigma^{2}+\nu^{2}}{\sqrt{\sigma^{2}\tau^{2}+\sigma^{2}\nu^{2}+\nu^{4}}}\;\exp\;\left\{-\frac{{\left[(\sigma^{2}+\nu^{2})(z-\bar{y})-\tau^{2}(z-\bar{z})\right]}^{2}}{2(\sigma^{2}\tau^{2}+\sigma^{2}\nu^{2}+\nu^{4})(\sigma^{2}+\nu^{2}-\tau^{2})}+\frac{{\left(\bar{z}-\bar{y}\right)}^{2}}{2(\sigma^{2}+\nu^{2}-\tau^{2})}\right\}.$$

At the equilibrium $\left(\bar{z},\bar{y})=(\theta ,\theta \right)$,

A 2 $$U(\bar{z}\pm k\sigma )=\frac{\sigma^{2}+\nu^{2}}{\sqrt{\sigma^{2}\tau^{2}+\sigma^{2}\nu^{2}+\nu^{4}}}\;\exp\;\left[-\frac{k^{2}\sigma^{2}(\sigma^{2}+\nu^{2}-\tau^{2})}{2(\sigma^{2}\tau^{2}+\sigma^{2}\nu^{2}+\nu^{4})}\right],$$

so that U(z)≈1 holds when

A 3 $$\frac{\left|\sigma^{2}+\nu^{2}-\tau^{2}\right|}{\tau^{2}+\nu^{2}+\nu^{4}\text{/}\sigma^{2}}\ll 1.$$

As parameters *ν*^2^, *σ*^2^ and *τ*^2^ are independent in the model, this condition is not necessarily satisfied, but if we add the assumption $\tau^{2}\approx \sigma^{2}+\nu^{2}$, U(z)≈1 is always true. The variance of preference distribution may increase if choosy individuals care little about the traits of the opposite sex. Moreover, sampling and observation errors affect both preference and trait variances, although these errors may differ between the sexes. Therefore, it is reasonable to assume that the variance of the preference distribution, *τ*^2^, increases when choosiness is mild (*ν*^2^ is large) and the trait of the decorative sex distributes widely (*σ*^2^ is large). In other words, the assumption $\tau^{2}\approx \sigma^{2}+\nu^{2}$ may not be unrealistic. Even if this assumption does not hold, the model results may not qualitatively change because $P_{M}{\bar{z}}_{M}+P_{L}{\bar{z}}_{L}=\bar{z}$ is always satisfied.

## Appendix B

From (2.15) and (2.16), the trait distribution in the next generation (breeding season) is

B 1 $$t(z^{\prime })=\frac{w(z^{\prime })\varphi (z^{\prime })}{\int w(z^{\prime })\varphi (z^{\prime })\text{d}z^{\prime }}=\frac{1}{\sqrt{2\pi }\sigma }\;\exp\;\left[-{\frac{\left[z^{\prime }-(1-q)\theta -q{\bar{z}}_{I})\right]}{2\sigma^{2}}}^{2}\right],$$

where

B 2 $${\bar{z}}_{I}=a{\bar{z}}_{M}+(1-a){\bar{z}}_{L}$$

and

B 3 $$q=\frac{\omega^{2}}{\kappa^{2}+\omega^{2}}.$$

Therefore, we have

B 4 $${\bar{z}}^{\prime }-\theta =q\{1-s[aR-(1-a)r]\}(\bar{z}-\theta )+qs[aR-(1-a)r](\bar{y}-\theta ).$$

Similarly, the preference distribution in the next generation (breeding season) is

B 5 $$p(y^{\prime })=\frac{1}{\sqrt{2\pi }\tau }\;\exp\;\left[-{\frac{\left(y^{\prime }-{\bar{y}}^{\prime }\right)}{2\tau^{2}}}^{2}\right],$$

where

B 6 $${\bar{y}}^{\prime }=b{\bar{z}}_{M}+(1-b){\bar{z}}_{L}.$$

Therefore, we have

B 7 $${\bar{y}}^{\prime }-\theta =\{1-s[bR-(1-b)r]\}(\bar{z}-\theta )+s[bR-(1-b)r](\bar{y}-\theta ).$$

## Appendix C

From (2.22), the equilibrium $\left(\bar{z},\bar{y})=(\theta ,\theta \right)$ is stable in a square area $a_{\min}\leq a\leq a_{\max}$ and $b_{\min}\leq b\leq b_{\max}$ when

C 1*a* $$\begin{array}{rl} b_{\max} & <\frac{1+sr}{s(R+r)}, \end{array}$$

C 1*b* $$\begin{array}{rl} b_{\max} & <a_{\min}+\frac{1}{qs(R+r)} \end{array}$$

C 1*c* $$\begin{array}{rl} \;\mathrm{and}\;b_{\min} & >\frac{2q}{1+q}a_{\max}-\frac{1+q-sr(1-q)}{s(1+q)(R+r)}, \end{array}$$

are satisfied. The square area $1-{\text{e}}^{-r}\leq a\leq 1$ and $1-{\text{e}}^{-r}\leq b<(1+sr)/s(R+r)$ satisfies this condition because 0 < *s* < 1, 0 < *q* < 1 and $1-{\text{e}}^{-r}-r{\text{e}}^{-r}>0$. As (1+sr)/s(R+r)>1, the equilibrium is stable, provided that $\bar{z}\leq {\bar{z}}_{I}\leq {\bar{z}}_{M}$ and $\bar{z}\leq {\bar{y}}^{\prime }\leq {\bar{z}}_{M}$ if $\bar{z}\leq {\bar{z}}_{M}$, or ${\bar{z}}_{M}\leq {\bar{z}}_{I}\leq \bar{z}$ and ${\bar{z}}_{M}\leq {\bar{y}}^{\prime }\leq \bar{z}$ if ${\bar{z}}_{M}<\bar{z}$.

## Appendix D

It is assumed that in each breeding season, mating partners are initially determined by the same process as in the cultural model, and that even if an individual does not have a mate initially, he/she may have a mate later, although his/her number of offspring decreases. When the fitness (relative number of offspring) of a decorative individual without a mate initially is 1  − *c* (0 < *c* ≤ 1), the fitness-weighted average trait of decorative individuals is

D 1 $$\begin{array}{rl} {\bar{z}}_{w} & =\frac{{\bar{z}}_{M}P_{M}+{\bar{z}}_{L}P_{L}(1-c)}{P_{M}+P_{L}(1-c)}\\ & =\bar{z}+\frac{c{\text{e}}^{-r}(1-{\text{e}}^{-r})}{1-c{\text{e}}^{-r}}({\bar{z}}_{M}-{\bar{z}}_{L}). \end{array}\}$$

As the trait is expressed in the decorative sex only, the proportion $h_{t}^{2}/2$ of the deviation from the average trait is inherited in the next generation where $h_{t}^{2}$ is the (narrow-sense) heritability of the trait ($0<h_{t}^{2}\leq 1$). Therefore, the average trait of decorative individuals in the next generation before viability selection is

D 2 $${\bar{z}}_{I}=\bar{z}+\frac{c{\text{e}}^{-r}(1-{\text{e}}^{-r})}{1-c{\text{e}}^{-r}}({\bar{z}}_{M}-{\bar{z}}_{L})\cdot \frac{h_{t}^{2}}{2}.$$

Rigorously speaking, when viability selection acts on the trait, it may be better to consider the deviation from the average *immature* trait to obtain the next generation trait, but this disagreement does not essentially change the results.

## Appendix E

As the proportion $\rho^{2}/(\rho^{2}+\omega^{2})$ of the variance in the modified trait *z* is caused by the genetic trait *x*, the average trait of decorative individuals in the next generation before viability selection (and trait modification) is

E 1 $$\begin{array}{rl} {\bar{x}}_{I} & =\bar{x}+\frac{\rho^{2}}{\rho^{2}+\omega^{2}}\frac{c{\text{e}}^{-r}(1-{\text{e}}^{-r})}{1-c{\text{e}}^{-r}}({\bar{z}}_{M}-{\bar{z}}_{L})\frac{h_{t}^{2}}{2}\\ & =\bar{x}+C(\bar{y}-\bar{z}), \end{array}\}$$

where

E 2 $$C=\frac{\rho^{2}}{\rho^{2}+\omega^{2}}\frac{c{\text{e}}^{-r}(1-{\text{e}}^{-r})}{1-c{\text{e}}^{-r}}\frac{h_{t}^{2}}{2}s(R+r).$$

Assuming that the variance of trait in the next generation before viability selection is *χ*^2^, the average trait in the next generation after viability selection (but before trait modification) is

E 3 $$\begin{array}{rl} {\bar{x}}^{\prime } & =\frac{\xi^{2}}{\xi^{2}+\chi^{2}}{\bar{x}}_{I}+\frac{\chi^{2}}{\xi^{2}+\chi^{2}}\theta \\ & =k[\bar{x}+C(\bar{y}-\bar{z})]+(1-k)\theta , \end{array}\}$$

where

E 4 $$k=\frac{\xi^{2}}{\xi^{2}+\chi^{2}}.$$

Then, the average trait in the next generation after viability selection and trait modification is

E 5 $$\begin{array}{rl} \bar{z} & =q{\bar{z}}_{I}+(1-q){\bar{x}}^{\prime }\\ & =q\{1-s[aR-(1-a)r]\}\bar{z}+qs[aR-(1-a)r]\bar{y}+(1-q)\{k[\bar{x}+C(\bar{y}-\bar{z})]+(1-k)\theta \}. \end{array}\}$$

The average preference in the next generation is the same as (2.20). Therefore, the recursive matrix is drawn

E 6 $$\begin{array}{rl} \left(\begin{array}{c} {\bar{z}}^{\prime }-\theta \\ {\bar{y}}^{\prime }-\theta \\ {\bar{x}}^{\prime }-\theta \end{array}\right) & =\left(\begin{array}{ccc} q\{1-s[aR-(1-a)r]\}-k(1-q)C & qs[aR-(1-a)r]+k(1-q)C & k(1-q)\\ 1-s[bR-(1-b)r] & s[bR-(1-b)r] & 0\\ -kC & kC & k \end{array}\right)\\ & \;\times \left(\begin{array}{c} \bar{z}-\theta \\ \bar{y}-\theta \\ \bar{x}-\theta \end{array}\right). \end{array}$$

As 0 < *k* < 1 and 0 < *C* < 1, the equilibrium $\left(\bar{z},\bar{y},\bar{x})=(\theta ,\theta ,\theta \right)$ is stable, provided both $1-{\text{e}}^{-r}\leq a\leq 1$ and $1-{\text{e}}^{-r}\leq b\leq 1$ are satisfied, i.e. $\bar{z}\leq {\bar{z}}_{I}\leq {\bar{z}}_{M}$ and $\bar{z}\leq {\bar{y}}^{\prime }\leq {\bar{z}}_{M}$ if $\bar{z}\leq {\bar{z}}_{M}$, or ${\bar{z}}_{M}\leq {\bar{z}}_{I}\leq \bar{z}$ and ${\bar{z}}_{M}\leq {\bar{y}}^{\prime }\leq \bar{z}$ if ${\bar{z}}_{M}<\bar{z}$.

## Appendix F

As in the second model (appendix D), assuming the fitness (relative number of offspring) of a decorative individual without a mate initially to be 1 − *c*, the fitness of a choosy individual with preference *y* is

F 1 $$\gamma (y)=\int \psi^{*}(z|y)t(z)\frac{1-c+c\pi (z)}{U(z)}\text{d}z$$

because the probability that a *y* choosy individual courts and mates with a *z* decorative individual is $\psi^{*}(z|y)t(z)/U(z)$ and the relative number of offspring of *z* individuals is π(z)+(1−c)[1−π(z)]=1−c+cπ(z). Therefore, the fitness-weighted average preference of choosy individuals is

F 2 $$\begin{array}{rl} {\bar{y}}_{w} & =\int yp(y)\gamma (y)\text{d}y\\ & =\bar{y}+\frac{\left(1-{\text{e}}^{-r})(1-c{\text{e}}^{-r}-cr{\text{e}}^{-r}\right)}{r(1-c{\text{e}}^{-r})}({\bar{z}}_{M}-{\bar{z}}_{L}), \end{array}\}$$

where we assume $\tau^{2}\approx \sigma^{2}+\nu^{2}$ (see appendix A). As the preference is expressed in the choosy sex only, the proportion $h_{p}^{2}/2$ of the deviation from the average preference is inherited in the next generation where $h_{p}^{2}$ is the (narrow-sense) heritability of the preference ($0<h_{p}^{2}\leq 1$). Therefore, the average preference of choosy individuals in the next generation is

F 3 $${\bar{y}}^{\prime }=\bar{y}+\frac{\left(1-{\text{e}}^{-r})(1-c{\text{e}}^{-r}-cr{\text{e}}^{-r}\right)}{r(1-c{\text{e}}^{-r})}({\bar{z}}_{M}-{\bar{z}}_{L})\frac{h_{p}^{2}}{2}.$$
